# Histone Deacetylase 1 and Sirtuin 1 Expression in Psoriatic Skin: A Comparison between Guttate and Plaque Psoriasis

**DOI:** 10.3390/life10090157

**Published:** 2020-08-21

**Authors:** Young-Ji Hwang, Jung-Im Na, Sang-Young Byun, Soon-Hyo Kwon, Seung-Hye Yang, Hyun-Sun Lee, Hye-Ryung Choi, Soyun Cho, Sang Woong Youn, Kyoung-Chan Park

**Affiliations:** 1 Department of Dermatology, Seoul National University Bundang Hospital, Seongnam 13620, Korea; derma_yj@naver.com (Y.-J.H.); jina1@snu.ac.kr (J.-I.N.); 1bsyoung@naver.com (S.-Y.B.); soonhyo17@gmail.com (S.-H.K.); seunghye84@naver.com (S.-H.Y.); hyhssky3@naver.com (H.-S.L.); hyeryung.choi@gmail.com (H.-R.C.); swyoun@snu.ac.kr (S.W.Y.); 2 Department of Dermatology, Seoul National University College of Medicine, Seoul 03080, Korea; sycho@snu.ac.kr; 3 Institute of Human-Environment Interface Biology, Medical Research Center, Seoul National University, Seoul 03080, Korea; 4 Department of Dermatology, Seoul National University Boramae Hospital, Seoul 07061, Korea

**Keywords:** histone deacetylase, sirtuin, epigenetics, psoriasis, guttate psoriasis, plaque psoriasis

## Abstract

Abnormal histone modification by histone deacetylases (HDACs), including HDAC1 and sirtuin 1 (SIRT1), has been reported to play an important role in the pathogenesis of psoriasis by altering cell proliferation, differentiation, and inflammation. However, findings on the expression level of HDACs in psoriatic skin lack consistency. We assessed the expression of HDAC1, SIRT1, p63, and proliferating cell nuclear antigen (PCNA) in skin tissues from 23 patients with psoriasis (15 with plaque psoriasis and eight with guttate psoriasis) and five healthy individuals using immunohistochemistry, and analyzed their associations with clinical phenotypes of the disease. The expression of HDAC1 and keratinocyte proliferative markers, such as p63 and PCNA significantly increased, whereas that of SIRT1 decreased in the basal layer (*p* < 0.05) of the patients with psoriasis compared to those in healthy controls. Among the patients with psoriasis, expression of HDAC1, p63, and PCNA was significantly higher in plaque psoriasis than in guttate psoriasis. There was no significant differences in the level of SIRT1 between the two clinical phenotypes. The findings of this study suggest that histone modifications are involved in the pathogenesis of psoriasis and may contribute to the formation of clinical phenotypes.

## 1. Introduction

Psoriasis is a common inflammatory disorder of the skin, characterized by excessive proliferation and abnormal differentiation of keratinocytes [1]. Psoriasis is classified into five morphological subtypes, among which plaque and guttate psoriasis are the most common clinical phenotypes [2]. Some studies have shown that plaque and guttate psoriasis are associated with Th1 cytokines and Th17-mediated immune responses, respectively [3,4]. However, few studies have attempted to determine the correlations of biochemical markers with clinical phenotypes.

The development of psoriatic lesions is known to involve complex interactions between genetic and environmental factors, which are not fully understood yet. Epigenetic modifications, changes in gene expression in the absence of alterations in DNA sequence [5], are emerging putative links between genetic and environmental factors in the pathogenesis of psoriasis [6]. Histone acetylation and deacetylation are essential parts of epigenetic regulation. Histone deacetylases (HDACs) remove acetyl groups from the lysine residues of histones, resulting in a more condensed chromatin structure and repressed gene transcription. HDAC proteins are grouped into four categories based on function and DNA sequence similarity. The acetylation of histones is primarily regulated by HDAC1, 2, and 3 proteins, which belong to the class I HDAC [7].

Inhibitors of HDACs have emerged as a possible treatment option for psoriasis because of their anti-inflammatory, anti-proliferative and anti-angiogenic effects [8,9,10,11]. There are controversies as to whether HDACs are upregulated in psoriasis. HDAC1 mRNA has been reported to be overexpressed in lesional skin and peripheral blood mononuclear cells (PBMC) of patients with psoriasis [12,13]. However, Ekman and Enerbӓck found no differences in HDAC activity and HDAC1, 2, and 3 protein expression levels between patients with psoriasis and healthy controls [14]. 

Sirtuin 1 (SIRT1) is a nicotinamide adenine dinucleotide (NAD+)-dependent class III HDAC, crucial for cell survival, metabolism, senescence, and stress response [15]. Similar to HDAC1, SIRT1 removes acetyl groups from histones, but it also has various non-histone targets including p53, nuclear factor-κB (NF-κB) and peroxisome proliferator-activated receptor- γ (PPAR-γ) [16]. The role of SIRT1 in psoriasis is opposite to that of HDAC1. SIRT1 exhibits anti-inflammatory [17,18] and anti-proliferative activity [19]. SIRT1 expression is reported to be decreased in fibroblasts and keratinocytes of lesional psoriatic skin [20,21,22,23]. However, only a few studies involving small sample sizes have examined the expression levels of SIRT1 in patients with psoriasis. 

In this study, we examined the expression of HDAC1, SIRT1, and the proliferation markers p63 and proliferating cell nuclear antigen (PCNA) in guttate and plaque psoriasis using immunohistochemistry to investigate whether they are dysregulated in psoriatic skin and if there is any difference between subtypes of psoriasis. 

## 2. Materials and Methods 

### 2.1. Patients

A total of 23 patients with psoriasis (8 with guttate psoriasis and 15 with plaque psoriasis), with a mean age of 45.6 ± 15.2 (range, 21–74) years, and five healthy volunteers were enrolled. The study was approved by the institutional review board (IRB No. B-1405/250-301) of the research institution. Both clinical and histopathological diagnoses were conducted, based on the following three major inclusion criteria: no local or systemic treatment for at least 4 weeks prior to entering the study; no significant infection or immune suppression; and no significant renal, hepatic, or other medical conditions. Patients were divided into either the guttate or the plaque group according to their clinical psoriasis phenotypes at the initial visits. Guttate psoriasis was defined as acute onset or reactivation of scattered, small plaque lesions of <1 cm in diameter; while patients with plaque psoriasis exhibited nummular and large plaques (≥1 lesion with a long-axis diameter of >5 cm). Punch biopsies were performed under local anesthesia. All samples were fixed using neutral buffered formalin and embedded in paraffin. Hematoxylin–eosin-stained sections were examined for pathological changes such as: (i) the degree of acanthosis and parakeratosis in the epidermis, and (ii) the degree of inflammation and angiogenesis in the dermis. These histopathological features were graded on a 4-point scale according to their prominence: 0, none; 1, mild; 2, moderate; and 3, marked. 

### 2.2. Immunohistochemical Staining

The expression of HDAC1, SIRT1, p63, and PCNA was compared between patients with psoriasis (plaque and guttate) and healthy controls using immunohistochemistry. Immunohistochemical staining was performed in 5 μm thick formalin-fixed paraffin-embedded tissue sections. Slides were deparaffinized in xylene and then dehydrated in a series of graded ethanol concentrations. Antigen retrieval was performed using a Trilogy solution (Cell Marque, Rocklin, CA, USA) and a pressure cooker. After blocking using normal goat serum and donkey serum, the tissue sections were incubated overnight at 4 °C with primary antibodies. Thereafter, the sections were washed and incubated with secondary antibodies and stained using DAPI (4,6-diamino-2-phenylindole). Images were obtained using a confocal laser scanning microscope (LSM710, Carl Zeiss, Jena, Germany) and ZEN 2011 microscope software (Carl Zeiss). Staining intensity was quantitated by averaging the values of three independent fields per section using ImageJ software (National Institutes of Health, Bethesda, MD, USA) [24]. Digital image analysis was performed as previously described [24,25]. Briefly, each digital image was processed by using the “color deconvolution” plugin. Color deconvolution involves isolation of the color information from histological red, green, and blue (RGB) images containing multiple stains. The area fraction of positive staining was computed for each epidermal compartment. 

### 2.3. Antibodies

Antibodies against HDAC1 (10E2, sc-81598), p63 (4A4, sc-8431), and PCNA (FL-261, sc-7907) were obtained from Santa Cruz Biotechnology, Inc. (Santa Cruz, CA, USA). Anti-SIRT1 antibody (E104, ab32441) was purchased from Abcam (Cambridge, UK). Secondary antibodies were Alexa Fluor^®^ 488 goat anti-mouse IgG (A11001, Molecular Probes^®^, Invitrogen, Carlsbad, CA, USA) and Alexa Flour^®^ 555 donkey anti-rabbit IgG (A31572, Molecular Probes^®^).

### 2.4. Statistical Analysis

Statistical analysis was performed using IBM SPSS Statistics Version 22 (Chicago, IL, USA). All data are presented as mean ± standard deviation (SD). The Mann–Whitney U-test and Kruskal–Wallis test were used to compare quantitative variables. For comparisons between qualitative variables, the Chi-squared test or Fisher’s exact test was used. Correlation analysis was performed using the Pearson correlation test or Spearman’s rank correlation test. Statistical significance was accepted at *p* < 0.05.

## 3. Results

The clinical and histopathological data of the patients with psoriasis enrolled in the study are presented in Table 1.

### 3.1. Expression of HDAC1, SIRT1, and Proliferative Markers in Patients with Psoriasis and Healthy Controls

**Figure 1:**
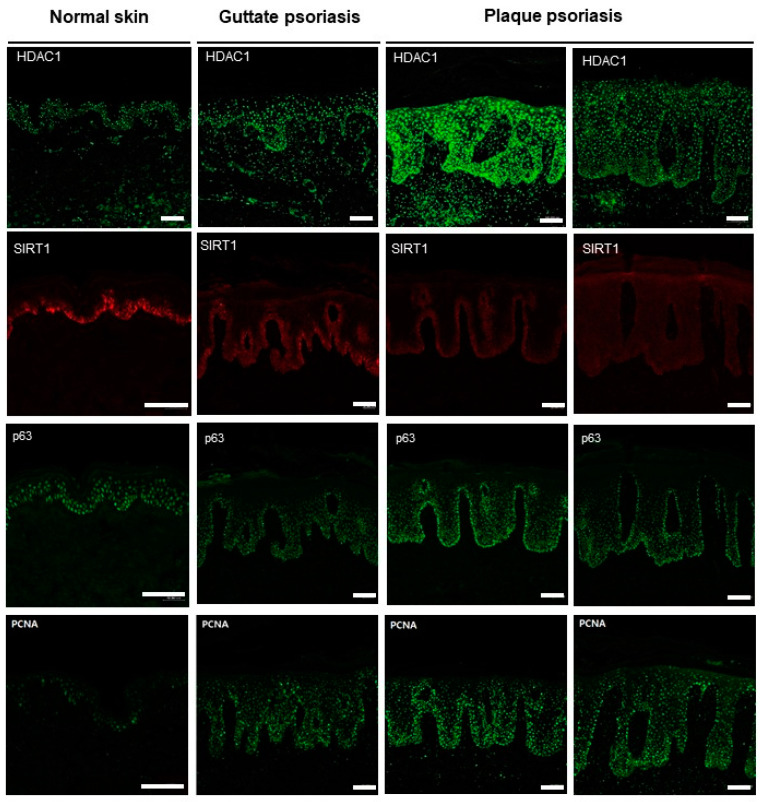
Expression of HDAC1, SIRT1, p63, and PCNA in guttate psoriasis, plaque psoriasis, and normal skin (Bars: 100 μm).

The expression of HDAC1, SIRT1, p63, and PCNA was compared between patients with psoriasis and healthy controls using immunohistochemistry (Table 2 and Figure 1). HDAC1 was expressed throughout the epidermis in both normal and psoriatic tissues but was significantly upregulated in psoriatic lesions (*p* < 0.05). As expected, the expression of proliferation biomarkers, PCNA and p63, was also significantly increased in the psoriatic skin (both *p* < 0.01). SIRT1 was only detected in the basal layer of epidermis in both psoriatic skin and healthy controls, and the staining intensity was significantly lower in psoriatic lesions.

The association between each marker and clinical and histopathological characteristics was analyzed. There was no significant correlation between HDAC1, SIRT1, or PCNA level with age, or disease duration. P63 showed a significant positive correlation with disease duration (r = 0.47, *p* < 0.05 *, Pearson correlation test), but not with age. The HDAC1 expression level was positively correlated with the degree of acanthosis, parakeratosis, and inflammation (*p* < 0.05, *p* < 0.05, *p* < 0.05, respectively, Spearman’s rank correlation test), but not with angiogenesis (*p* = 0.0654). SIRT1 level did not show a significant association with histopathological characteristics. P63 and PCNA expression levels were positively correlated with acanthosis (*p* < 0.05, *p* < 0.01, respectively, Spearman’s rank correlation test) but not with inflammation, parakeratosis, or angiogenesis.

### 3.2. Expression of HDAC1, SIRT1, and Proliferative Markers in Patients with Guttate and Plaque Psoriasis

Next, we compared the expression of HDAC1, SIRT1, p63, and PCNA in guttate and plaque psoriasis. The expression of HDAC1, p63, and PCNA was significantly higher in the plaque psoriasis group than in the guttate psoriasis group (*p* < 0.01, *p* < 0.01, and *p* < 0.05, respectively; Table 3 and Figure 1). HDAC1 was expressed throughout the epidermis in both plaque and guttate psoriasis. SIRT1 showed no significant difference between plaque and guttate psoriasis (Table 3 and Figure 1). 

## 4. Discussion

Although the introduction of biologics has greatly improved the treatment of psoriasis, patients’ satisfaction with existing therapies remains modest [26]. Therefore, new therapeutic targets need to be explored. Abnormal histone modifications are considered as potential therapeutic targets for psoriasis; however, the epigenetic profile of the disease has not been fully elucidated. In this study, we observed a significant increase in HDAC1 expression in psoriatic lesions compared to healthy controls. In addition, HDAC1 expression levels were higher in plaque psoriasis than in guttate psoriasis. Compared to guttate psoriasis, plaque psoriasis has a chronic disease course and tends to be more resistant to treatment. The fact that HDAC1 expression levels were higher in plaque psoriasis suggests that HDAC1 could have a more important role in chronic and severe psoriasis. The present study is the first to compare the expression of HDACs in different clinical phenotypes of psoriasis; thus, it could provide a partial explanation for the previous conflicting results on HDAC expression in vivo. Increased expression of HDAC1, together with p63 and PCNA biomarkers, could contribute to the formation of clinical phenotypes of psoriasis. 

SIRT1 is another epigenetic therapeutic target for psoriasis. Some studies have reported that deacetylation of NF-κB by SIRT1 downregulates tumor necrosis factor (TNF)-α and other proinflammatory cytokines [27,28], while also inhibiting the cell cycle mediator retinoblastoma (Rb)/E2F1 and increasing cell death of keratinocytes [19,29]. Based on these studies, SIRT1 activators have been investigated as therapeutic agents for psoriasis; however, the treatment outcomes have been inconsistent [30]. In a phase IIa clinical study on the efficacy of a SIRT1 activator (SRT2104) in psoriasis, 35% patients showed good to excellent histological improvement, but did not demonstrate a dose-dependent response according to histology endpoints [31]. In our study, we observed decreased expression of SIRT1 in patients with psoriasis than in healthy controls, and there was no difference between guttate and plaque psoriasis. Further studies are required to determine the clinical phenotypes of patients who respond to the SIRT1 activator and those who do not.

A small sample size and imbalance of sample size between plaque and guttate psoriasis is the limitation of this study. Compared to plaque psoriasis, it was much more difficult to recruit subjects with guttate subtype because guttate psoriasis is less common and transient. The lack of quantitative measures such as Western blot is another limitation of this study. Further studies with larger sample sizes and including other clinical subtype will facilitate a better understanding of the role of epigenetic modification in the pathogenesis of psoriasis and the development of clinical subtypes.

## 5. Conclusions

A decrease in SIRT1 and an increase in HDAC1 expression levels were observed in psoriatic skin, along with p63 and PCNA upregulation. Furthermore, there were significant differences in the expression levels of HDAC1 as well as p63 and PCNA between plaque and guttate psoriasis. Our findings suggest that epigenetic modifications are involved in the pathogenesis of psoriasis and may contribute to the formation of clinical phenotypes.

## Figures and Tables

**Table 1:** Clinical and histopathological characteristics of psoriatic patients.

**Clinical Data**		
Age (years)	Mean ± SD	45.6 ± 15.2
	Median	45
	Range	21–74
Sex	Male	12 (52.2%)
	Female	11 (47.8%)
	M:F ratio	1.1:1
Duration	Mean ± SD	31 ± 39.4
(months)	Median	12
	Range	0.5–144
Clinical phenotype	Guttate	8 (34.8%)
	Plaque	15 (65.2%)
	Guttate: Plaque ratio	1:1.86
**Histopathological Data**		
Acanthosis	Mild	6 (26.1%)
	Moderate	10 (43.5%)
	Marked	6 (26.1%)
Parakeratosis	Mild	11 (47.8%)
	Moderate	9 (39.1%)
	Marked	1 (4.4%)
Inflammation	Mild	10 (43.5%)
	Moderate	10 (43.5%)
	Marked	3 (13.0%)
Angiogenesis	Mild	8 (34.8%)
	Moderate	13 (56.5%)
	Marked	2 (8.7%)

**Table 2:** Comparison of immunohistochemical staining intensity between healthy controls and psoriasis patients, and results of statistical assessments.

	Healthy Controls (n = 5)	Psoriasis Patients (n = 23)	*p*-Value ^a^
HDAC1	21.82 ± 6.28	40.47 ± 17.73	*p* < 0.05 *
SIRT1	51.15 ± 8.67	31.02 ± 12.51	*p* < 0.01 *
P63	19.29 ± 3.70	37.83 ± 11.93	*p* < 0.01 *
PCNA	20.22 ± 3.86	42.02 ± 17.62	*p* < 0.01 *

HDAC1, histone deacetylase 1; SIRT1, sirtuin 1; PCNA, proliferating cell nuclear antigen. ^a^ Mann–Whitney U test or Student’s *t*-test between healthy controls and psoriasis patients. * denote statistically significant difference at *p* < 0.05.

**Table 3:** Comparison of immunohistochemical staining intensity between guttate, and plaque psoriasis patient groups.

	Guttate Psoriasis (n = 8)	Plaque Psoriasis (n = 15)	*p*-Value ^a^
HDAC1	26.27 ± 15.09	48.04 ± 14.27	*p* < 0.01 *
SIRT1	36.19 ± 13.92	28.26 ± 11.20	*p* = 0.152
p63	27.56 ± 3.98	43.31 ± 11.10	*p* < 0.01 *
PCNA	26.89 ± 14.46	50.09 ± 13.51	*p* < 0.05 *

HDAC1, histone deacetylase 1; SIRT1, sirtuin 1; PCNA, proliferating cell nuclear antigen. ^a^ Mann–Whitney U test or Student *t*-test between patients with guttate psoriasis and plaque psoriasis. * denote statistically significant difference at *p* < 0.05.

## References

[1] Lowes M.A., Bowcock A.M., Krueger J.G. (2007). Pathogenesis and therapy of psoriasis. Nature.

[2] Griffiths C.E., Christophers E., Barker J.N., Chalmers R.J., Chimenti S., Krueger G.G., Leonardi C., Menter A., Ortonne J.P., Fry L. (2007). A classification of psoriasis vulgaris according to phenotype. Br. J. Dermatol..

[3] Christophers E. (2008). Explaining phenotype heterogeneity in patients with psoriasis. Br. J. Dermatol.

[4] Choe Y.B., Hwang Y.J., Hahn H.J., Jung J.W., Jung H.J., Lee Y.W., Ahn K.J., Youn J.I. (2012). A comparison of serum inflammatory cytokines according to phenotype in patients with psoriasis. Br. J. Dermatol..

[5] Bird A. (2007). Perceptions of epigenetics. Nature.

[6] Brooks W.H., Le Dantec C., Pers J.O., Youinou P., Renaudineau Y. (2010). Epigenetics and autoimmunity. J. Autoimmun..

[7] Newbold A., Matthews G.M., Bots M., Cluse L.A., Clarke C.J., Banks K.M., Cullinane C., Bolden J.E., Christiansen A.J., Dickins R.A. (2013). Molecular and biologic analysis of histone deacetylase inhibitors with diverse specificities. Mol. Cancer Ther..

[8] Leoni F., Zaliani A., Bertolini G., Porro G., Pagani P., Pozzi P., Dona G., Fossati G., Sozzani S., Azam T. (2002). The antitumor histone deacetylase inhibitor suberoylanilide hydroxamic acid exhibits antiinflammatory properties via suppression of cytokines. Proc. Natl. Acad. Sci. USA.

[9] Falkenberg K.J., Johnstone R.W. (2014). Histone deacetylases and their inhibitors in cancer, neurological diseases and immune disorders. Nat. Rev. Drug. Discov..

[10] Saunders N., Dicker A., Popa C., Jones S., Dahler A. (1999). Histone deacetylase inhibitors as potential anti-skin cancer agents. Cancer Res..

[11] Kwon H.J., Kim M.S., Kim M.J., Nakajima H., Kim K.W. (2002). Histone deacetylase inhibitor FK228 inhibits tumor angiogenesis. Int. J. Cancer.

[12] Tovar-Castillo L.E., Cancino-Diaz J.C., Garcia-Vazquez F., Cancino-Gomez F.G., Leon-Dorantes G., Blancas-Gonzalez F., Jimenez-Zamudio L., Garcia-Latorre E., Cancino-Diaz M.E. (2007). Under-expression of VHL and over-expression of HDAC-1, HIF-1alpha, LL-37, and IAP-2 in affected skin biopsies of patients with psoriasis. Int. J. Dermatol..

[13] Zhang P., Su Y., Zhao M., Huang W., Lu Q. (2011). Abnormal histone modifications in PBMCs from patients with psoriasis vulgaris. Eur. J. Dermatol..

[14] Ekman A.K., Enerback C. (2016). Lack of preclinical support for the efficacy of histone deacetylase inhibitors in the treatment of psoriasis. Br. J. Dermatol..

[15] Guarente L., Franklin H. (2011). Epstein Lecture: Sirtuins, aging, and medicine. N. Engl. J. Med..

[16] Serravallo M., Jagdeo J., Glick S.A., Siegel D.M., Brody N.I. (2013). Sirtuins in dermatology: Applications for future research and therapeutics. Arch. Dermatol. Res..

[17] Lee J.H., Moon J.H., Lee Y.J., Park S.Y. (2017). SIRT1, a Class III Histone Deacetylase, Regulates LPS-Induced Inflammation in Human Keratinocytes and Mediates the Anti-Inflammatory Effects of Hinokitiol. J. Invest. Dermatol..

[18] Jung Y.J., Lee J.E., Lee A.S., Kang K.P., Lee S., Park S.K., Lee S.Y., Han M.K., Kim D.H., Kim W. (2012). SIRT1 overexpression decreases cisplatin-induced acetylation of NF-kappaB p65 subunit and cytotoxicity in renal proximal tubule cells. Biochem. Bioph. Res. Commun..

[19] Blander G., Bhimavarapu A., Mammone T., Maes D., Elliston K., Reich C., Matsui M.S., Guarente L., Loureiro J.J. (2009). SIRT1 promotes differentiation of normal human keratinocytes. J. Invest. Dermatol..

[20] Sestito R., Madonna S., Scarponi C., Cianfarani F., Failla C.M., Cavani A., Girolomoni G., Albanesi C. (2011). STAT3-dependent effects of IL-22 in human keratinocytes are counterregulated by sirtuin 1 through a direct inhibition of STAT3 acetylation. FASEB J..

[21] Becatti M., Barygina V., Emmi G., Silvestri E., Taddei N., Lotti T., Fiorillo C. (2016). SIRT1 activity is decreased in lesional psoriatic skin. Intern. Emerg. Med..

[22] Fan X., Yan K., Meng Q., Sun R., Yang X., Yuan D., Li F., Deng H. (2019). Abnormal expression of SIRTs in psoriasis: Decreased expression of SIRT 1-5 and increased expression of SIRT 6 and 7. Int. J. Mol. Med..

[23] Becatti M., Barygina V., Mannucci A., Emmi G., Prisco D., Lotti T., Fiorillo C., Taddei N. (2018). Sirt1 Protects against Oxidative Stress-Induced Apoptosis in Fibroblasts from Psoriatic Patients: A New Insight into the Pathogenetic Mechanisms of Psoriasis. Int. J. Mol. Sci..

[24] Jensen E.C. (2013). Quantitative analysis of histological staining and fluorescence using Image. Anat. Rec. (Hoboken).

[25] Brianezi G., Minicucci E.M., Marques M.E., Miot H.A. (2013). Evaluation epidermal p53 immunostaining by digital image analysis. Skin Res. Technol..

[26] Florek A.G., Wang C.J., Armstrong A.W. (2018). Treatment preferences and treatment satisfaction among psoriasis patients: A systematic review. Arch. Dermatol. Res..

[27] Yeung F., Hoberg J.E., Ramsey C.S., Keller M.D., Jones D.R., Frye R.A., Mayo M.W. (2004). Modulation of NF-kappaB-dependent transcription and cell survival by the SIRT1 deacetylase. EMBO J..

[28] Zhu X., Liu Q., Wang M., Liang M., Yang X., Xu X., Zou H., Qiu J. (2011). Activation of Sirt1 by resveratrol inhibits TNF-α induced inflammation in fibroblasts. PLoS ONE.

[29] Lee J.H., Kim J.S., Park S.Y., Lee Y.J. (2016). Resveratrol induces human keratinocyte damage via the activation of class III histone deacetylase, Sirt1. Oncol. Rep..

[30] McLaughlin F., La Thangue N.B. (2004). Histone deacetylase inhibitors in psoriasis therapy. Cur. Drug Targets Inflamm. Allergy.

[31] Krueger J.G., Suárez-Fariñas M., Cueto I., Khacherian A., Matheson R., Parish L.C., Leonardi C., Shortino D., Gupta A., Haddad J. (2015). A Randomized, Placebo-Controlled Study of SRT2104, a SIRT1 Activator, in Patients with Moderate to Severe Psoriasis. PLoS ONE.

